# St. George's Hospital and King Edward's Fund

**Published:** 1912-05-25

**Authors:** Savile Crossley, John G. Griffiths

**Affiliations:** Honorary Secretary. King Edward's Hospital Fund for London, 7 Walbrook, E.C.; Honorary Secretary. King Edward's Hospital Fund for London, 7 Walbrook, E.C.


### THE EDITOR'S LETTER-BOX.

### St. George's Hospital and King Edward's Fund.

LETTER FROM THE HONORARY SECRETARIES OF THE KING'S FUND.

TO THE EDITOR OF "THE TIMES."

Sir,—It is only within the last few days that the King's Fund has received from St. George's Hospital any intimation of the reasons for their inability to accept the conclusion arrived at by the Fund in March of last year on the subject of the financial relation between that hospital and its Medical School. We notice that in his letter of the 14th inst. the treasurer of the hospital defends only two out of the six statements which we described as inaccuracies, and we propose therefore to confine ourselves to these two.

In the first place, the treasurer of the hospital denies that any intimation was conveyed to St. George's that the Fund could not accept the change in the accounts of the hospital without investigation. In support of this he quotes a letter from the Fund dated May 6, 1909. But he has quoted only half the letter, omitting the paragraphs which contained the intimation to which we referred. The remainder of the letter is as follows :—

"It appears to the Committee that if the payments mentioned in your letter do not come within the terms of the first proviso (*i.e.* are not specific payments for definite work done in respect of the treatment of patients), the proposal to make them out of the general funds would not be admissible, but that if they do come within those terms the statistical tables for 1908 and previous years do not represent the true cost of the treatment of patients as required by the Revised Uniform System.

"It is the practice of the Fund (in the absence of evidence to the contrary) to accept the accounts submitted by a hospital and the chairman's certificate on the subject of the Medical School. But the Fund is placed in a position of some difficulty if there is a want of consistency between these either in the same year or over a series of years."

There was therefore a clear intimation that the Fund could not accept without investigation a change which rendered the accounts of succeeding years inconsistent with one another as regards payments to the Medical School. These paragraphs were referred to in the Fund's letter of October 13, 1910, in which the Distribution Committee invited the deputation to which the treasurer alludes.

In the second place, he defends the statement that the Fund changed its ground from the question of a contribution to the School to one of extravagance. In support of this he gives an extract from the Fund's report, which merely described, not a change in the object of the inquiry, but a change in the direction in which to seek reliable evidence. It therefore becomes necessary to explain the reason for this change in method, a reason which the Distribution Committee in courtesy refrained from mentioning in their final report.

As the question to be decided was whether or not the hospital proportion of the cost of the laboratories was excessive and involved an indirect contribution to the school, the committee had previously inquired into the basis of the apportionment, and had been informed by the deputation that the charges to the hospital were based on the amount the hospital would have had to pay for maintaining the laboratories necessary for its purpose, if there were no school to share the cost of them and consequently to bear a portion of the cost. The Fund, in its letter of November 16, 1910, expressed the opinion that this basis of apportionment was unsound, and asked for a reapportionment based on the relative value received by each participant. In reply to this the authorities of St. George's submitted, not a revised apportionment, but a fresh arrangement of the figures, based on the new method of apportionment, but giving exactly the same result as that based on the old.

It was after this that the committee fell back upon the alternative method of comparing the cost of the work at St. George's with the cost at other hospitals, as evidence as to whether the apportionment, the basis of which they had these reasons for doubting, did actually produce a reasonable result. This line of inquiry led them, as has been stated, to the conclusion that the payment was so far in excess of the average that the apportionment could not be accepted as correct, but that it involved an indirect contribution to the school—indirect in the sense of being not a deliberate grant to medical education, but an overpayment for work done by the school in respect of the treatment of patients.

This conclusion of the sub-committee, which was expressly stated not to be intended as any reflection on the *bona fides* of those responsible for the apportionment, was adopted both by the full Distribution Committee and by the General Council of the Fund.

We are unable to appreciate the grounds for the intervention in this matter of St. George's Hospital Medical School, the hon. treasurer of which writes officially to *The Times* a letter published on the 15th inst. We are not aware that any one on behalf of King Edward's Hospital Fund has expressed any doubt whatever as to the good administration of the funds placed at the disposal of the school; it is the hospital authorities, not the administrators of the Medical School, upon whom the duty is placed of carefully controlling expenditure on the investigation of the ailments of patients who are being treated in the hospital.

We may say, however, that in suggesting that there is an inconsistency between our statement that the cost at St. George's was "about 180 per cent. above the average of other hospitals" while "the report of the committee said two and a half times," the hon. treasurer of the school is himself quoting only half a statement. For the report states that the cost at St. George's Hospital worked out at two and a half times the average (or 150 per cent. above the average), if St. George's was included, or slightly over two and three-quarter times (or 175 per cent. above) if St. George's was excluded.

We are, sir, your obedient servants,

SAVILE CROSSLEY, } Honorary

JOHN G. GRIFFITHS, } Secretaries.

King Edward's Hospital Fund for London,
7 Walbrook, E.C., May 20.

### The Plans of the Sheffield Royal Hospital.

*To the Editor of* The Hospital.

